# Screening of novel alkaloid inhibitors for vascular endothelial growth factor in cancer cells: an integrated computational approach

**DOI:** 10.5808/gi.20068

**Published:** 2021-03-15

**Authors:** Shah Md. Shahik, Asma Salauddin, Md. Shakhawat Hossain, Sajjad Hossain Noyon, Abu Tayab Moin, Shagufta Mizan, Md. Thosif Raza

**Affiliations:** 1 Molecular Biology Department, AFC Agro Biotech Ltd., Dhaka 1212, Bangladesh; 2 Bioinformatics Division, Disease Biology and Molecular Epidemiology Research Group (dBme), Chattogram 4202, Bangladesh; 3 Department of Genetic Engineering and Biotechnology, Faculty of Biological Sciences, University of Chittagong, Chattogram 4331, Bangladesh

**Keywords:** alkaloids, angiogenesis, cancer, drug-likeness, molecular docking, vascular endothelial growth factor, virtual screening

## Abstract

Vascular endothelial growth factor (VEGF) is expressed at elevated levels by most cancer cells, which can stimulate vascular endothelial cell growth, survival, proliferation as well as trigger angiogenesis modulated by VEGF and VEGFR (a tyrosine kinase receptor) signaling. The angiogenic effects of the VEGF family are thought to be primarily mediated through the interaction of VEGF with VEGFR-2. Targeting this signaling molecule and its receptor is a novel approach for blocking angiogenesis. In recent years virtual high throughput screening has emerged as a widely accepted powerful technique in the identification of novel and diverse leads. The high-resolution X-ray structure of VEGF has paved the way to introduce new small molecular inhibitors by structure-based virtual screening. In this study using different alkaloid molecules as potential novel inhibitors of VEGF, we proposed three alkaloid candidates for inhibiting VEGF and VEGFR mediated angiogenesis. As these three alkaloid compounds exhibited high scoring functions, which also highlights their high binding ability, it is evident that these alkaloids can be taken to further drug development pipelines for use as novel lead compounds to design new and effective drugs against cancer.

## Introduction

Cancer is a multifactorial disease that gets influenced by several factors including genetic change, lifestyle, viral infection, bacterial infection and epigenetic effects. Cancer causes an elevated physical toll along with amplified psychological stress that disrupts homeostasis [1]. In terms of fatality, cancer undoubtedly falls in the category of diseases that accounts for high death cases and stands second following cardiac diseases. Every year about 1 in 6 deaths occur due to cancer globally which is about 10 million deaths per year [2,3]. Cancer’s effect on the older population (aged 70 or above) is perniciously leading to a high fatality rate which was projected to be 14.4% in older males and 9.6% in older females in 2019 [4].

Cancer has seven hallmarks which include: selective growth and proliferative advantage, altered stress response favoring overall survival, apoptosis, invasion and metastasis, metabolic rewiring/reprogramming, an abetting microenvironment, and immune modulation [5]. When it comes to aiding both normal and abnormal cell proliferation, angiogenesis plays a vital role [6]. Angiogenesis refers to construction of new capillary blood vessels from pre-existing blood vessels to supply sufficient molecular oxygen, nutrients and other essentials to the proliferating cells. Through the process of angiogenesis, cellular waste and debris are also removed hence angiogenesis or vascularization has a significant role in maintaining cell viability, development, and proliferation [7-9]. Tumor cell proliferation is pronouncedly dependent on angiogenesis because when tumors are devoid of nascent blood vessels to supply them with the necessary factors required for proliferation, they remain benign and ultimately die from necrosis and apoptosis [7,10,11]. Angiogenesis also amplifies the cancer state by providing the abnormal cells with a network to carry out metastasis and corresponding secondary infection [12]. However, several factors either upregulate or downregulate angiogenesis hence, the process is susceptible to being either positively or negatively altered by activators and inhibitors [7,13].

Among the activators of angiogenesis, vascular endothelial growth factors (VEGFs) play a fundamental role as signaling proteins that stimulate new blood vessel formation by vasculogenesis and angiogenesis throughout our lifetime [14,15]. Usually these signaling proteins bind to specific VEGF receptors which then elicit a cellular response of vessel formation [16].

The VEGF proteins are made up of five known sub-families namely VEGF-A (the highly conserved founding member), VEGF-B, VEGF-C, VEGF-D (also known as c-Fos‒induced growth factor) and the viral VEGF-Es encoded by strains D1701, NZ2, and NZ7 of the parapoxvirus Orf (which causes pustular dermatitis) [17]. VEGF-A is the prototypical member of a family of associated growth factors that includes placental growth factor [17]. The different classes of VEGFs carry out different functions in relation to angiogenesis [18]. The VEGF class that gains the most attention in terms of research is the VEGF-A class as it is thought to be the primary class of VEGF that promotes systemic primary blood vessel development [17]. The discrete functions of VEGF-A that have been identified are follows: increasing endothelial cell migration, increasing permeability of blood vessels, and maintenance of uniform neovascularization [17]. VEGF-B takes embryonic vasculogenesis to completion in combination with VEGF-A [19]. VEGF-C was found to uniquely contribute to lymphomagenesis as it binds to the VEGF receptor (VEGFR)-3 receptor and VEGF-D plays a role in pulmonary angiogenesis through binding to the VEGFR-3 receptor as well. There are also two other classes of VEGF namely VEGF-E and VEGF-F [17]. VEGF-E is encoded by viruses that synergistically along with virus particles such as IL-10 helps wound healing as found in mice and for the VEGF-F case, it is usually isolated and found in snake venom [20].

As far as the mechanism goes for VEGF binding, VEGF-A can bind with either of the corresponding receptors VEGFR-1 or VEGFR-2 located on the surface of the endothelial cells [21]. However, VEGF-A most commonly binds to the VEGFR-2 to stimulate vessel growth [22]. The other receptor VEGFR-3 is specific to another class of VEGF (VEGF-C) and it is thought that the pathway upon binding that receptor stimulates the proliferation of lymphatic cells [21]. All of these receptors are tyrosine kinase receptors which causes dimerization and activation by transphosphorylation which ultimately results in vessel formations [23].

Anti-angiogenic drugs and in particular anti-VEGF agents have entered the clinical armamentarium against cancer. However, a number of complications in terms of vascular events have been found succeeding treatment. The vascular endothelial growth factor signaling pathway (VSP) inhibitors include antibodies that work both extracellularly and intracellularly on VEGF and VEGFR, respectively. VSP inhibitors have possibilities of eliciting damage to endothelial lining due to depleted endothelial cell turnover [24]. Inhibitor Mediated vascular anomalies also include arterial and/or venous thrombosis, and renal vascular injury [25]. Bevacizumab retains the highest frequency of bleeding complications, in particular epistaxis, hemoptysis, and gastrointestinal bleeding. Although a higher incidence of severe hemorrhages has not been consistently demonstrated during the treatment with bevacizumab, mild bleeding episodes appear clearly increased in the experimental arm of most trials. Trials with other small-molecule tyrosine kinase inhibitors like sunitinib or sorafenib showed an overall lower rate of bleeding complications, but still significantly higher than the control arm in many cases [26].

The mechanisms of bleeding induced by anti-VEGF agents are complex and not yet fully clarified: the main hypothesis is that VEGF could promote endothelial cell survival and integrity in the adult vasculature and its inhibition may decrease the renewal capacity of damaged endothelial cells [27]. Management of bleeding in patients treated with anti-VEGF agents is a challenging task because this complication is at least in part inherent to the efficacy of the drug and because there is also an increased risk of thrombosis, both arterial and venous. So far, only a few preliminary data are available on a strategy to prevent hemorrhage and thrombotic events [28]. However, previous studies have concluded that the deleterious effects of anti-VEGF drugs are not overt during the first stages of administration because of VEGF’s intrinsic roles relevant to vascular protection [29]. If subsidiary vascular thrombosis and other vascular complications can be minimized, VEGF inhibitors, if not of the conventional kind, can still be favorable in depleting the prognosis of tumor cells through blocking angiogenesis [30].

VEGF molecules have become a choice of interest for cancer therapy among scientists. Using virtual screening (VS) to find inhibitors against VEGFs from libraries of small molecules like alkaloids can be a good approach to inhibit angiogenesis in recent years [31]. VS refers to a computer-based technique used to identify drugs from libraries of small molecules that may be highly likely to interact with a certain enzyme or protein based receptor.

The aim of this study was to select alkaloids having similar binding capabilities as VEGF inhibitors to propose possible therapeutic candidates against tumor angiogenesis which might minimize vascular complications manifested by the current drugs. We curated a library of alkaloids to select ligands having similar binding affinity to that of anti-VEGF drugs. Since alkaloids have minimal side effects and are easier to extract, this study aimed to provide a preliminary list of potential alkaloids that can be used to develop highly effective therapeutics against VEGF molecules that can work against cancer.

## Methods

### Protein retrieval

The X-ray crystallographic protein structure of the major regulators of angiogenesis, VEGF-A (302aa, PDB Code: 1VPF), VEGF-B (207aa, PDB Code: 2C7W), VEGF-C (419aa, PDB Code: 2X1X), VEGF-D (354aa, PDB Code: 2XV7) were retrieved from the RCSB Protein Data Bank in PDB format which were going to be used as targets for carrying out the docking experiments. Resolutions of 2.5 Å, 2.48 Å, 3.1 Å, and 2.9 Å were employed for VEGF-A, VEGF-B, VEGF-C, and VEGF-D, respectively.

### Prediction of active site

In proteins, active sites are clefts formed by specific combinations of amino acids that facilitate the binding of ligands to a target protein often initiating or blocking a chain of reactions. Identification of the residues that make up the active site has a range of applications in molecular docking and de novo drug designing [32]. Computed atlas of surface topography of proteins (CASTp) was used in active site residue analysis [33,34]. CASTp works using Swiss-Prot mapping method as well as Online Mendelian Inheritance in Man (OMIM) mapping method to prognosticates specific amino acid positioning within a protein surface [35,36].

### Ligand retrieval and preparation

Initially, more than 300 alkaloid compounds were retrieved from different literature sources as control ligands for the purpose of inhibiting VEGFs based on their natural sources, few or no side effects as therapeutic agents and so on. These alkaloids were acquired from PubChem [37] and ZINC databases were used as ligands [38]. The compounds were downloaded in sdf or structural data file format and then converted to pdb format using OPEN Babel converter [39]. In the next step, these ligands were energy minimized and torsion angle of these molecules were changed for flexibility or freedom of movement. Currently, available known drugs were also retrieved and optimized in silico to be used as a ligand molecule for molecular docking analysis.

### Molecular docking

Structure-based virtual screening was done using molecular docking as it is a viable and effective process for the identification of hits or potential drugs and thus plays a major role in enhancing the lead recognition stage of the pharmaceutical sectors. VS by docking was selected because it is free, easy to use and can take advantage of numerous core processors in addition to having much more orderly search of the probable energy surfaces. VS was performed against the energy minimized models of VEGF-A, VEGF-B, VEGF-C, and VEGF-D using Autodock to carry out automated docking of ligand molecules to their macromolecular receptors. Autodock creates the three binding energy phases: intermolecular energy, internal energy of ligand, and torsional free energy [40]. The final docked energy is determined from the summation of intermolecular energy and internal energy of the ligand. Autodock tools were employed to construct the input pdbqt file for VEGF-A, VEGF-B, VEGF-C, and VEGF-D and also to set up the size and the center of the grid box. All water molecules, cofactors, and ligands were removed from the protein structure and then checked for polar hydrogen atoms in the macromolecules. Afterward, torsion bonds of the ligands were selected. The binding energy of macromolecules coordinate were evaluated by a three dimensional grid box of 80 × 40 × 80 (num.grid points in xyz) and grid center 5.958 × 2.623 × 28.642 (xyz-coordinates), 40 × 60 × 44 (num.grid points in xyz) and grid center ‒43.699 × ‒24.709 × ‒0.6 (xyz-coordinates), 76 × 50 × 70 (num.grid points in xyz) and grid center -34.28 × 2.751 × 13.25 (xyz-coordinates) and 30 × 60 × 50 (num.grid points in xyz) and grid center ‒30.389 × ‒36.541 × ‒6.255 (xyz-coordinates) were created for VEGF-A, VEGF-B, VEGF-C, and VEGF-D respectively(unit of the dimensions, Å). The bound ligand and actual target docking site was represented based on the calculation of the grid map and the final docking complex were visualized in BIOVIA Discovery Studio Visualizer v12.1.0.15350 [41].

### Bioavailability and ADME/Tox test

Absorption, distribution, metabolism, excretion, and toxicity (ADME/Tox) explain in detail the kinetics of drug exposure to the body tissues and pharmacological effects of the compounds. ADME/Tox was assessed with the help of an online server, preADMET [42]. Besides ADME, drug toxicity and its side effects of the compounds, a major concern, was estimated using OSIRIS program [43] and ADME/Tox filter with FAF-Drug-2 [44]. ADME/Tox filter with FAF-Drug-2 also eradicates PAINS (Pan Assay Interference Compounds) which provides further refining steps in the selection process. They provide weak options for drug development but can provide data that in isolation may be evocative of a particular and optimizable fit for potential drugs.

## Results and Discussion

**Fig. 1.**
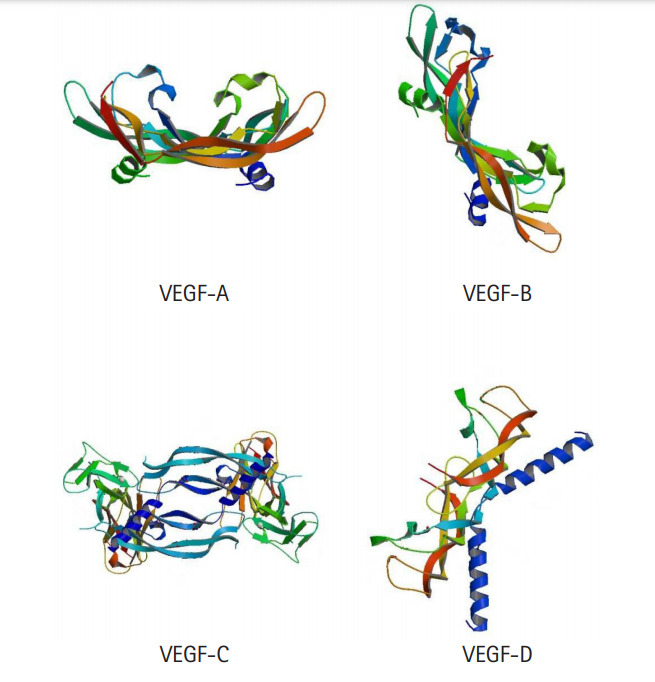
Crystal structure of VEGF-A, VEGF-B, VEGF-C, and VEGF-D. VEGF, vascular endothelial growth factor.

Cancer occupies the maximum landscape among the diseases and disorders that are found to be in frequent prevalence, due to its mortality rates as well as multiple other collateral risk factors. Often, cancer is detected at a stage beyond the scopes of cure by therapeutics because of its ability to blend in well with normal cells, which is why conventional treatment measures fail to provide a permanent cure for cancer patients [45,46]. Discovering and developing novel therapeutics against different types of cancer is quite difficult, merely because of the seven hallmarks that cancer imposes [46]. However, like multiple other diseases, different types of cancers have common clinical manifestations across individuals and if these mechanisms and common manifestations can be addressed using drugs, developing effective and consistent treatment methods against cancer will be possible. Among the hallmarks of cancer, angiogenesis is of immense importance and is common in all types of cancers [47]. As angiogenesis is regulated by VEGF-mediated signaling pathways, blocking VEGF action could stop angiogenesis and by extension, halt the growth of cancer cells, which is why VEGF is a suitable target for cancer therapy [48]. Different VEGF families with their receptors and their respective functions are listed in Table 1 and the crystal 3D structures are shown in Fig. 1. In this study, to scrutinize the effectiveness of alkaloids against cancer therapy in comparison with existing drugs that act upon VEGF blocking, we analyzed multiple alkaloids to identify potential inhibitors of multiple VEGFs using computational approaches of protein-ligand docking. Because VS is a widely followed procedure for de novo drug design, it helps in identifying a library of potential inhibitors which can further be analyzed in terms of binding affinity using molecular docking.

### Analysis of active site

Possible binding sites for different VEGFs were identified using the CASTp server [34]. The amino acid residues involved in binding pockets are given in Supplementary Table 1. The possible binding residues that were found to be involved in the interaction with lead inhibitors. As calculated by CASTp the binding pocket of VEGF-A, VEGF-B, VEGF-C, and VEGF-D has a volume of 122.264˚ A, 90.134˚ A, 291.758˚ A, and 14.779˚ A and surface area of 161.609, 149.220, 239.334, and 44.37 respectively.

### Ligand preparation

**Fig. 2.**
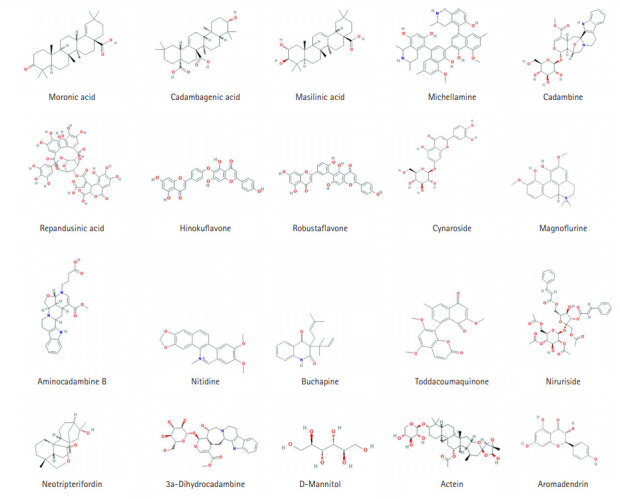
The 2D structure of 20 alkaloid compounds.

Based on ADME properties through VS of 20 compounds were shortlisted to create the ligand library with potential candidates (Fig. 2). We screened the selected compounds and selected those which exhibited preferable binding energy clusters [49]. Protein-substrate binding gives us insights into prediction and ranking of compounds on the basis of their binding and interactions [50].

### Molecular docking analysis

**Fig. 3.**
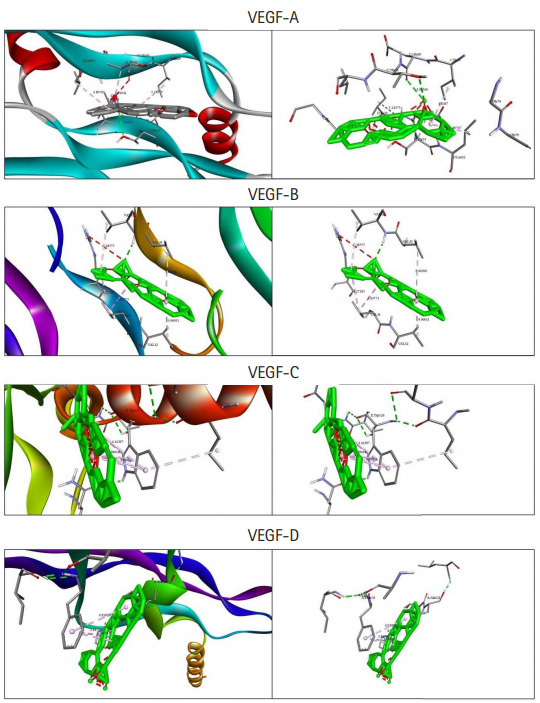
Graphical representation of molecular docking of VEGF-A, VEGF-B, VEGF-C, and VEGF-D with Moronic acid (green color indicate Moronic acid and the dashed-line indicate bonds). VEGF, vascular endothelial growth factor.

Among the currently available drugs against VEGFs, Ponatinib showed the highest binding free energy (Table 2) which were −10.8 kcal/mol, −9.4 kcal/mol, −10.0 kcal/mol, and −9.1 kcal/mol against VEGF-A, VEGF-B, VEGF-C, and VEGF-D, respectively. Hydrogen bonds, electrostatic bonds, and hydrophobic bonds were majorly formed with VEGFs and the interaction sites are shown in Table 3. Because ponatinib, among the drugs that are commonly used for angiogenesis inhibition exhibited a preferable and considerable binding affinity, it was used as the positive control. Now, although ponatinib is a widely used drug, it isn’t devoid of side effects. The most common adverse effects that can occur due to consistent ponatinib usage are thrombocytopenia and pancreatitis. To avoid these additional drawbacks, our aim was to look for alternative therapeutic compounds with minimum to no side effects. From the 20 ligands, we selected potential candidates for VEGF inhibition in Table 4. Among three ligands: moronic acid, cadambagenic acid, and masilinic acid exhibited higher binding energies with subsequent VEGFs which were more than those shown by ponatinib (Table 5). During docking with VEGF-A, Moronic acid formed three conventional hydrogen bonds with C:Glu30, C:Thr31, and D:Thr31 and three hydrophobic bonds with C:Ile29, D:Ile29, and D:Leu32. Most of the bonds were formed in the active site of the protein. With VEGF-B Moronic acid formed a hydrogen bond with A:Val32 and six hydrophobic bonds with the site A:Val31, A:VAL32, B:ARG29, B:VAL31, and B:VAL32. These bonds were formed on the same active site similar to that of ponatinib; however, the binding energy generated from moronic acid‒VEGF-B binding was higher than that generated from the binding with ponatinib. Docking with VEGF-C, moronic acid generated only six hydrophobic bonds at E:Trp126. Finally with VEGF-D five hydrophobic bonds at A:Ala121, A:Phe131, and A:Pro135 were formed. These bind strongly with the active site residues of the VEGFs signaling molecule so it can’t readily bind with its receptor (Fig. 3) and consequently block the signal transduction for angiogenesis. We also assessed their stability and observed that all bonds were of very short distance that indicates the intense bonding strength.

### ADME/Tox test analysis

ADME/Tox test analysis was carried out to assess the molecular properties, carcinogenicity and oral toxicity of the selected alkaloid candidates for VEGF inhibition (Tables 6 and 7). Their permeability to different cells and the blood brain barrier were also analyzed because all in all, these are the major stakeholders in drug discovery. The results obtained from these assessments validated the use of these alkaloids in effecting cancer treatment.

### Conclusion

In this study, we adapted in silico approaches of drug discovery to identify potential alkaloids that can prove effective in cancer treatment through VEGF receptor blocking hence obstructing angiogenesis. Through VS and molecular docking analysis, we were able to find three potential alkaloids that showed considerable binding affinity to VEGF active sites. Although in vivo interactions with VEGF active sites might differ from those observed in silico, our findings and propositions can give a head start to further investigations and experiments both in vitro and in vivo for developing anticancer drugs specific to blocking angiogenesis through the conventional pipeline.

## Figures and Tables

**Table 1.** Different types of VEGFs and their functions

VEGF family member	Receptor	Function
VEGF-A	VEGFR-1	Angiogenesis
	VEGFR-2	Vasodilation
		Chemotactic
VEGF-B	VEGFR-1	Embryonic angiogenesis
VEGF-C	VEGFR-2	Lymphangiogenesis
	VEGFR-3	
VEGF-D	VEGFR-2	Lymphangiogenesis
	VEGFR-3	
VEGF-E	VEGFR-2	Angiogenesis

VEGF, vascular endothelial growth factor.

**Table 2.** Docking results of different drugs with VEGFs

No.	Drug	VEGF-A	VEGF-B	VEGF-C	VEGF-D
1	Iclusig, Ponatinib	‒10.8	‒9.4	‒10.0	‒9.1
2	Votrient, Pazopanib	‒10.5	‒9.2	‒8.8	‒7.3
3	Adriamycin, Adriamycin	‒10.0	‒9.8	‒9.8	‒8.5
4	Cometriq, Cabozantinib	‒9.7	‒8.8	‒8.6	‒7.4
5	Inlyta, Axitinib	‒9.3	‒8.4	‒8.8	‒7.9
6	Stivarga, Regorafenib	‒9.0	‒9.6	‒9.3	‒8.3
7	Cabometyx, Cabozantinib	‒9.0	‒8.3	‒9.5	‒7.7
8	Lenvima, Lenvatinib	‒8.4	‒7.6	‒7.3	‒6.5
9	Sutent, Sunitinib	‒8.3	‒7.4	‒7.7	‒7.6
10	Nexavar, Sorafenib	‒8.3	‒8.5	‒9.2	‒7.4

AutoDock Vina scores are in kcal/mol. VEGF, vascular endothelial growth factor.

**Table 3.** Nonbonding interactions of ponatinib with VEGFs

VEGF	Bonds Donor (distance, Å) acceptor (bond type)		
	Hydrogen bond	Electrostatic bond	Hydrophobic bond
VEGF-A	B:SER50:HG (1.868) :LIG1:O (HB)	A:GLU64:OE2 (3.661) :LIG1:F (E, Halogen) A:GLU64:OE2 (3.271) :LIG1:F (E, Halogen) :LIG1:F (4.893) A:GLU64:OE2 (E) :LIG1:N (5.345) A:GLU64:OE2 (E) A:GLU64:O (3.635) :LIG1:F (Halogen)	A:ASN62:C,O;A:ASP63:N (3.949) :LIG1 (A-Pi-Stacked) B:LYS48 (3.955) :LIG1 (A) :LIG1 (3.952) B:LYS48 (Pi-A)
VEGF-B	B:VAL32:HN (2.244) :LIG1:O (HB) :LIG1:O (3.375) B:GLU30:O (HB) B:VAL31:CA (3.061) :LIG1:O (CHB) :LIG1:C (3.631) B:GLU30:O (CHB) :LIG1:C (3.397) B:CYS57:O (CHB)	A:VAL32:O (3.150) :LIG1:F (Halogen)	A:VAL31 (5.118) :LIG1 (A) B:VAL32 (4.166) :LIG1 (A)
VEGF-C	E:GLY141:HN (1.964) :LIG1:F (HB; Halogen) E:PRO155:CA (1.964) :LIG1:F (CHB; Halogen)	E:ASP139:OD2 (3.342) :LIG1:F (E, Halogen) E:ASP139:OD2 (3.488) :LIG1:F (E, Halogen) :LIG1:F (3.852) E:ASP139:OD2 (E) :LIG1:N (4.030) E:ASP139:OD2 (E) :LIG1:N (4.658) E:ASP139:OD2 (E) :LIG1:N (4.531) R:ASP276:OD1 (E) E:PRO155:O (3.173) :LIG1:F (Halogen) E:PRO155:O (3.119) :LIG1:F (Halogen)	E:PHE151 (5.429) :LIG1:C (Pi-A)
VEGF-D		:LIG1:F (5.304) A:GLU119:OE2 (E) A:PHE132:O (3.193) :LIG1:F (Halogen) :LIG1:N (4.027)A:PHE131 (E)	:LIG1:C (4.676) A:LYS133 (A) :LIG1:C (3.626) A:PRO135 (A)

Pose predicted by AutoDockVina where, HB, conventional hydrogen bond; CHB, carbon hydrogen bond; E, electrostatic; A, alkyl; Pi-A , pi-alkyl; A-Pi, amide-pi. VEGF, vascular endothelial growth factor.

**Table 4.** Docking results of different alkaloids with VEGFs

No.	Alkaloid	VEGF-A	VEGF-B	VEGF-C	VEGF-D
1	Moronic acid	‒12.9	‒13.2	‒11.9	‒12.2
2	Cadambagenic acid	‒12.5	‒12.2	‒11.5	‒11.5
3	Masilinic acid	‒12.4	‒12.6	‒11.5	‒12.0
4	Nortripterifordin	‒10.4	‒9.7	‒10.1	‒10.0
5	Michellamine	‒10.2	‒10.1	‒9.9	‒8.9
6	Cadambine	-10	‒9.6	‒9.3	‒7.8
7	Repandusinic acid	‒9.8	‒10.4	‒9.3	‒9.2
8	3a-Dihydrocadambine	‒9.6	‒9.0	‒9.4	7.4
9	Hinokiflavone	‒9.6	‒8.9	‒9.1	‒8.1
10	Robustaflavone	‒9.4	‒8.8	‒9.0	‒8.0

AutoDock Vina scores are in kcal/mol. VEGF, vascular endothelial growth factor.

**Table 5.** Molecular docking nonbonding interactions of moronic acid with VEGFs

VEGF	Bonds Donor (distance, Å) acceptor (bond type)	
	Hydrogen bond	Hydrophobic bond
VEGF-A	d:LIG1:O (3.095) C:GLU30:O (HB)	C:ILE29 (4.848) d:LIG1 (A)
	d:LIG1:O (2.954) C:THR31:OG1 (HB)	D:LEU32 (4.897) d:LIG1 (A)
	D:THR31:CA (3.185) d:LIG1:O (CHB)	d:LIG1 (5.243) D:ILE29 (A)
VEGF-B	A:VAL32:HN (2.226) d:LIG1:O (HB)	A:VAL31 (5.410) d:LIG1 (A)
		A:VAL32 (4.752) d:LIG1 (A)
		B:ARG29 (5.031) d:LIG1 (A)
		B:VAL31 (3.847) d:LIG1 (A)
		B:VAL31 (4.271) d:LIG1 (A)
		B:VAL32 (4.060) d:LIG1 (A)
VEGF-C		E:TRP126 (4.423) d:LIG1 (Pi-A)
		E:TRP126 (3.690) d:LIG1 (Pi-A)
		E:TRP126 (3.961) d:LIG1 (Pi-A)
		E:TRP126 (4.740) d:LIG1 (Pi-A)
		E:TRP126 (4.445) d:LIG1 (Pi-A)
		E:TRP126 (3.799) d:LIG1 (Pi-A)
VEGF-D		A:ALA121 (3.871) d:LIG1 (A)
		A:PRO135 (5.167) d:LIG1 (A)
		A:PHE131 (3.786) d:LIG1 (Pi-A)
		A:PHE131 (4.910) d:LIG1 (Pi-A)
		A:PHE131 (3.847) d:LIG1 (Pi-A)

VEGF, vascular epithelial growth factor; HB, conventional hydrogen bond; CHB, carbon hydrogen bond; A, alkyl; Pi-A, pi-alkyl.

**Table 6.** ADME prediction of final selected 10 alkaloids using pre-ADMET tool

No.	Compound	Human intestinal absorption (HIA, %)	Caco-2 cell permeability (nm/s)	MDCK cell permeability (nm/s)	Skin permeability (logKp, cm/h)	Blood brain barrier penetration (C.brain/C.blood)
1	Moronic acid	97.629	22.270	0.043	‒1.96099	4.35022
		(well absorbed)	(middle)	(low)		(high)
2	Cadambagenic acid	94.671	21.010	0.044	‒2.90457	2.72322
		(well absorbed)	(middle)	(low)		(high)
3	Maslinic acid	84.065	21.302	0.805	‒5.13957	0.258554 (middle)
		(well absorbed)	(middle)	(low)		
4	Nortripterifordin	95.204	21.983	112.722	‒3.06612	2.51705
		(well absorbed)	(middle)	(middle)		(high)
5	Michellamine	90.663	20.059	0.043	‒3.17237	2.32226
		(well absorbed)	(middle)	(low)		(high)
6	Cadambine	67.555	3.851	0.054	‒5.17586	0.0374348
		(medium absorbed)	(middle)	(low)		(low)
7	Repandusinic acid	00.000	15.834	0.043	‒2.63834	0.0277558
		(no absorbed)	(middle)	(low)		(low)
8	3a-Dihydrocadambine	52.205	6.134	0.073	‒5.11884	0.035656
		(medium absorbed)	(middle)	(low)		(low)
9	Hinokiflavone	86.954	7.156	0.084	‒3.36300	0.280203 (middle)
		(well absorbed)	(middle)	(low)		
10	Robustaflavone	81.196	12.043	0.043	‒3.45363	0.122688 (middle)
		(well absorbed)	(middle)	(low)		

ADME properties showed that these compounds are good lead molecules.

**Table 7.** Toxicity of final selected 10 alkaloids using OSIRIS Property Explorer

No.	Alkaloid	Toxicity effect			
		M, mutagenic	T, tumorigenic	I, irritant	R, reproductive
1	Moronic acid	No	No	No	No
2	Cadambagenic acid	No	No	No	No
3	Maslinic acid	No	No	No	No
4	Nortripterifordin	No	No	Yes	No
5	Michellamine	No	Yes	No	No
6	Cadambine	No	No	No	No
7	Repandusinic acid	No	No	-	-
8	3a-Dihydrocadambine	No	No	No	No
9	Hinokiflavone	No	No	No	Yes
10	Robustaflavone	No	No	-	-

## Supplementary Materials

Supplementary Table 1. The residue around active site of the protein was predicted by using CASTp
