# Supplementary material for: Screening of novel alkaloid inhibitors for vascular endothelial growth factor in cancer cells: an integrated computational approach
Source: Genomics Inform. 2021 Mar 15;19(1):e6. doi: 10.5808/gi.20068 (PMC8042301; doi:10.5808/gi.20068)
Supplement: Supplementary Table 1. — The residue around active site of the protein was predicted by using CASTp [file gi-20068-suppl.pdf]

Supplementary Table 1. The residue around active site of the protein was predicted by using CASTp

VEGF	Area (SA)	Volume (SA)	Sequence
VEGF-A	161.609	122.264	A:GLY59, A:CYS60, A:CYS61, A:ASN62, A:ASP63, A:GLU64, A:LEU66, A:GLU67, A:CYS68, A:LYS107, B:ASP34, B:ILE35, B:PHE36, B:ILE46, B:PHE47, B:LYS48, B:SER50
VEGF-B	149.220	90.134	A:LEU35, A:THR36, A:VAL37, A:LEU39, A:MET40, A:THR42, A:ALA44, A:LYS45, A:LEU47, A:ILE80, A:MET82, A:GLY91, A:GLU92, A:MET93
VEGF-C	239.334	291.758	R:TYR137, R:ILE138, R:THR139, R:GLU140, R:ASN141, R:LYS144, R:THR145, R:VAL146, R:SER189, R:TYR190, R:SER193, R:VAL218, R:VAL219, R:GLY220, R:TYR221, R:ARG222, R:TYR224, R:GLU251, R:LEU252, R:ASN253, R:GLU284
VEGF-D	44.737	14.779	A:VAL118, A:VAL120, A:GLU123, A:LEU124, A:TYR160, A:VAL181, A:LYS182, A:VAL183, A:ALA184

CASTp, computed atlas of surface topography of proteins; VEGF, vascular endothelial growth factor.
